# Diffusion Kurtosis Imaging Detects Microstructural Changes in the Brain after Acute Alcohol Intoxication in Rats

**DOI:** 10.1155/2017/4757025

**Published:** 2017-01-18

**Authors:** Xi-ran Chen, Jie-ying Zeng, Zhi-Wei Shen, Ling-mei Kong, Wen-bin Zheng

**Affiliations:** Department of Radiology, The Second Affiliated Hospital, Shantou University Medical College, Shantou, Guangdong, China

## Abstract

The aim of this study was to test the technical feasibility of diffusion kurtosis imaging (DKI) in the brain after acute alcohol intoxication. Diffusion tensor imaging (DTI) and DKI during 7.0 T MRI were performed in the frontal lobe and thalamus before and 30 min, 2 h, and 6 h after ethyl alcohol administration. Compared with controls, mean kurtosis values of the frontal lobe and thalamus first decreased by 44% and 38% within 30 min (*p* < 0.01 all) and then increased by 14% and 46% at 2 h (frontal lobe, *p* > 0.05; thalamus, *p* < 0.01) and by 29% and 68% at 6 h (frontal lobe, *p* < 0.05; thalamus, *p* < 0.01) after acute intake. Mean diffusivity decreased significantly in both the frontal lobe and the thalamus at various stages. However, fractional anisotropy decreased only in the frontal lobe, with no detectable change in the thalamus. This demonstrates that DKI possesses sufficient sensitivity for tracking pathophysiological changes at various stages associated with acute alcohol intoxication and may provide additional information that may be missed by conventional DTI parameters.

## 1. Introduction

Acute alcohol exposure may directly or indirectly affect the brain given many neurological changes such as blood flow, electrical brain activity, and memory [1–3]. Early medical and rehabilitative intervention may reduce more severe secondary damage to the brain, but both CT and conventional MRI usually fail to detect macroscopic and microscopic evidence of brain injury in acute alcohol exposure [4]. Thus, there is much interest in developing advanced neuroimaging techniques to detect and characterize microstructural changes that occur in the brain due to acute alcohol exposure [5–7].

In our previous study, we showed that diffusion tensor imaging (DTI) can detect microscopic tissue damage in the brain after acute alcohol intoxication based on fractional anisotropy (FA) and apparent diffusion coefficient values [8, 9]. DTI measurements are based on the assumption of a Gaussian displacement probability distribution of water molecules due to water self-diffusion, such as water in isotropic liquid media [10]. However, diffusion of water molecules in most biological tissues, especially brain tissues, is restricted by barriers, such as cellular membranes, which cause diffusion to deviate substantially from a Gaussian form [11, 12], making DTI a limited indicator of complexity. Therefore, new imaging methods based on non-Gaussian diffusion models, such as diffusion kurtosis imaging (DKI), may assess microstructural complexity more accurately than DTI, especially in gray matter [13–15]. Moreover, DKI datasets generally include DTI datasets as part of the total measurements [16].

The aims of this study were as follows: (1) to investigate microstructural abnormalities in the frontal lobe and thalamus of rat brains after acute alcohol intoxication using DKI metrics and DTI metrics, (2) to determine whether DKI provides additional information about underlying structural abnormalities compared with DTI, and (3) to investigate the kinetics of structural and functional changes in the brain at various stages following alcohol administration.

## 2. Materials and Methods

### 2.1. Animal Models

Thirty-five sexually mature (8- to 10-week-old) male Sprague-Dawley rats (200–250 g, from the Laboratory Animal Center of Shantou University Medical College, Shantou, Guangdong, China) were divided into 4 groups: 3 acute ethyl alcohol (EtOH) intoxication groups (30 min, 2 h, and 6 h; *n* = 10 for each group) and 1 control group (*n* = 5). Rats in the acute EtOH intoxication groups received an intragastrically injection of EtOH (Hongxing Erguotou wine, 56% vol, Beijing, China) at a dose of 15 mL/kg and were then examined every minute after EtOH administration for loss of the righting reflex to ensure acute alcohol intoxication [17]. The control group received 15 mL/kg of saline by intragastrically injection before MRI examination. The study protocol was approved by the ethics committee of Shantou University Medical College.

### 2.2. Imaging

All rats were scanned in a 7.0 T (Agilent VnmrJ 3 Imaging, USA) animal MR machine using a surface coil with a diameter of 30 mm. Rats in the 3 subgroups of acute EtOH intoxication were imaged separately at 30 min, 2 h, and 6 h after EtOH administration. The animals were anesthetized with 4% isoflurane mixed with 1 L/min oxygen and maintained with 2% isoflurane.

A 3-slice (axial, sagittal, and coronal) scout was used to ensure that the brain was in the proper position. T2-weighted imaging was obtained using 2D rapid acquisition with a fast spin-echo sequence using a 2000 ms repetition time (TR) and 5 slices at 2 mm thickness with a 0.2 gap and an in-plane resolution of 128 × 96 um. The T2-weighted imaging was used to position the DKI field of view.

Diffusion kurtosis imaging was obtained with a fast spin-echo multislice sequence, together with an in-plane resolution of 280 × 260 um and a TR/TE of 2000/36 ms at 2 mm thickness with a 0.2 gap, 2 averages, 4 shots, and 16 kzero. Two* b*-values (1000 s/mm^2^ and 2000 s/mm^2^) were applied to 30 directions following acquisition of the image at* b* = 0 s/mm^2^. Scan time for DKI was approximately 33 min.

Shimming was very important to MR image quality. Therefore, we only selected images that achieved a line width of less than 20 Hz in repeated shimming attempts.

### 2.3. Histology

After all images were obtained, animals were immediately killed for histology. Animals received an overdose of chloral hydrate and were perfused transcardially with 4% paraformaldehyde in 0.1 M phosphate buffer. Brains were removed and fixed with 4% paraformaldehyde for 48 h. After fixation, brains were embedded in paraffin, and contiguous 5 *μ*m sections at the level of the frontal lobe and thalamus were cut on a microtome (Rm 2016, LEICA, Germany). Sections were then stained with hematoxylin and eosin (HE).

### 2.4. Data Processing

For image analysis, MK, FA, and MD maps were processed with MATLAB (The Mathworks, Natick, MA, USA) and MRIcro software (Neuropsychology Lab, Columbia, SC, USA). Besides, because DKI datasets generally include DTI datasets as part of the total measurements, the FA and MD values were calculated by using part of the DKI model in equation (using only* b*-value = 1,000 s/mm^2^) [18, 19].

Regions of interest (ROIs) included the thalamus and frontal lobe (including precentral area and striate cortex). There were two reasons that we chose frontal lobe and thalamus as our regions of interest. On the one hand, because of the high sensitivity of 7.0 T animal MR machine and the high requirement about the shimming, we just chose one slice. On the other hand, these areas are more vulnerable to acute alcohol intoxication [8]. Frontal lobe plays a major role in cognitive functions such as working memory, decision-making, motor controlling, and language [20]. As the central relay station of the brain, the thalamus has mutual projections to the cerebral cortex, which also has a vital role in neuropsychological performance such as attention, concentration, and information processing [21, 22]. Damage to the frontal lobe and thalamus is associated with clinical sequelae and cognitive impairment.

**Figure 1:**
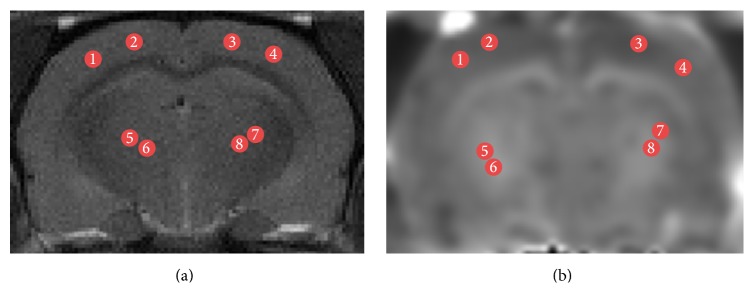
Illustration of ROIs on T2WI (a) and MK (b) maps for a representative rat on coronal slices. Regions shown are frontal lobe (1–4) and thalamus (5–8).

The average MK, FA, and MD values were calculated bilaterally on the left and right sides in the ROIs (Figure 1). Every ROI was tested four times to obtain the mean value and every region of interest was about 5 mm^2^.

### 2.5. Statistical Analysis

Each of the measured parameters (MD, FA, and MK) was expressed as mean ± standard deviation. Data analysis was conducted using SPSS 17.0 (SPSS Inc., Chicago, IL, USA) with a one-way ANOVA to compare groups. A level of *p* < 0.05 was considered statistically significant.

## 3. Results

**Figure 2:**
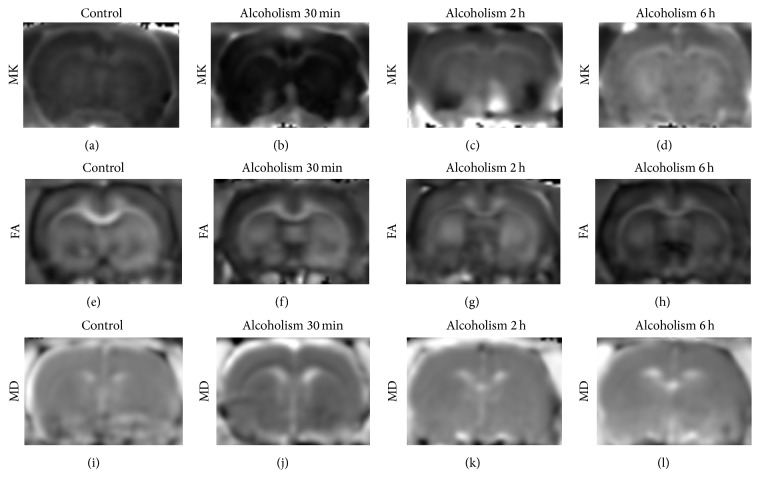
Representative MK, FA, and MD maps from controls and EtOH-treated rats. (a–d) MK values in the control group and in rats with alcohol intoxication at 30 min, 2 h, and 6 h; (e–h) FA values in the control group and in rats with alcohol intoxication at 30 min, 2 h, and 6 h; (i–l) MD values in the control group and in rats with alcohol intoxication at 30 min, 2 h, and 6 h.

**Figure 3:**
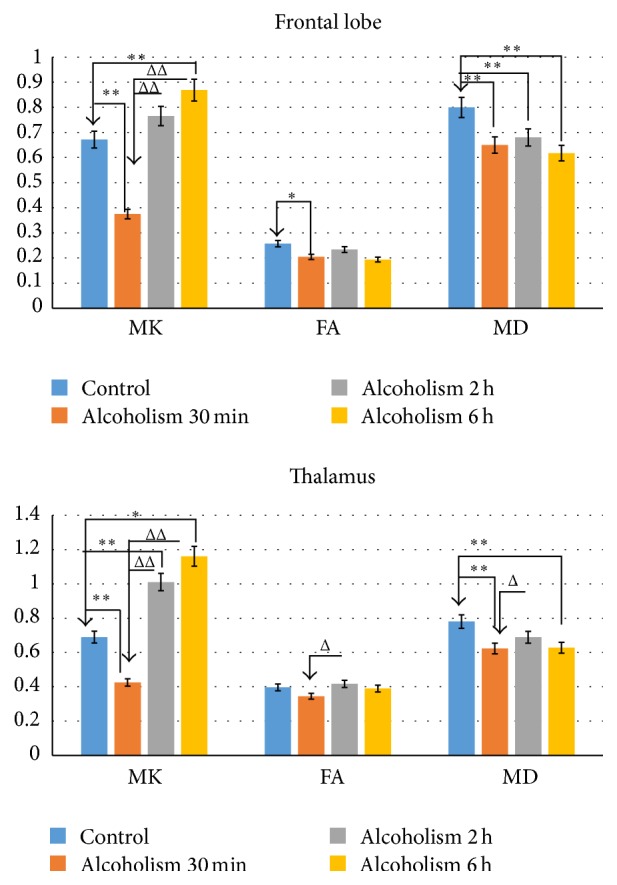
Changes in MK, FA, and MD values for frontal lobe and thalamus between controls and EtOH-treated rats. ^*∗*^Statistical significance was based on comparison with controls. ^Δ^Statistical significance was based on comparison with alcoholism 30 min. ^*∗*, Δ^*P* < 0.05 was considered to indicate a statistically significant difference. ^*∗∗*, ΔΔ^*P* < 0.01 was considered to indicate a clear statistically significant difference.

No obvious signal intensity abnormalities were observed in the T2-weighted images of all rats, whereas differences were detected in MK, MD, and FA parameters in the frontal lobe and thalamus (Figure 2). Comparisons of MK, MD, and FA values in different ROIs obtained from pre- and post-alcohol administration are reported in Figure 3 and Table 1. Compared with controls, MK decreased by 44% and 38% in the frontal lobe and thalamus at 30 min (frontal lobe: *p* < 0.01; thalamus: *p* < 0.01) and increased by 29% and 68% at 6 h (frontal lobe: *p* < 0.01; thalamus: *p* < 0.01). At 2 h, the observed increase (14% and 46%) did not reach statistical significance in the frontal lobe (frontal lobe: *p* > 0.05; thalamus: *p* < 0.01). Compared with 30 min after acute alcohol administration, MK increased by 104% and 138% in the frontal lobe and thalamus at 2 h and increased by 132% and 173% at 6 h (all: *p* < 0.01).

Compared with controls, a significant decrease in FA was observed in the frontal lobe at 30 min (*p* < 0.05) after acute alcohol administration, but the trend in reduced FA at the 2 h and 6 h time points was not significant (*p* > 0.05). No statistically significant differences were observed for the FA values in the thalamus at any of the 3 time points (*p* > 0.05). MD showed decreases both in the frontal lobe and thalamus at each of the 3 time points except at 2 h in the thalamus (2 h in the thalamus: *p* > 0.05; others: *p* < 0.01). Compared with 30 min after acute alcohol administration, a significant increase in FA and MD was only observed in the thalamus at 2 h (*p* < 0.05).

No statistically significant difference was observed for the MK, FA, and MD values between 2 h and 6 h after acute alcohol administration.

**Figure 4:**
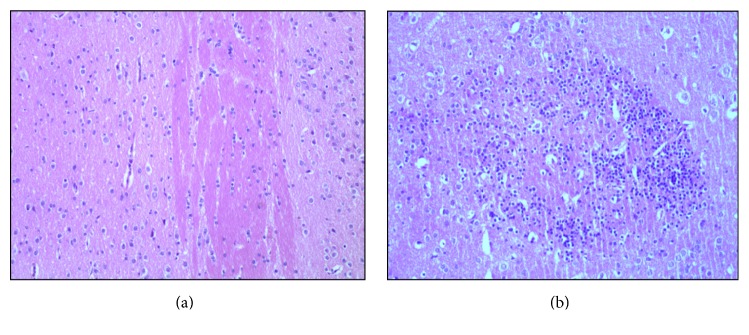
HE stains of representative acute alcohol intoxication rats and a sham rat. (a) Control rat; (b) acute alcohol intoxication rats at 6 h.

In the control group, HE staining revealed normal pericapillary spaces and intact blood vessel walls within the frontal lobe and thalamus. In addition, no erythrocytes were observed within the vascular lumina. Neurons exhibited normal morphology with clear round nuclei and a constant nucleus-cytoplasm ratio (Figure 4(a)). For alcohol-administered rats, HE staining mainly revealed neurons with edema and arranged in a disordered pattern (Figure 4(b)).

## 4. Discussion

There was a steady reduction in MD in the frontal lobe and thalamus at each of the observed time points after EtOH administration. This phenomenon has been well documented in our prior investigations [8, 9]. Changes in MD value are helpful for distinguishing and identifying the type of edema in lesions. A reduced MD suggests cytotoxic edema, whereas an increased MD indicates vasogenic edema [8, 9, 23]. Therefore, the observed decreased MD value 6 h after EtOH administration was indicative of cytotoxic edema, which is consistent with our HE staining results.

In contrast, no appreciable change in FA was observed in the frontal lobe and thalamus at the 3 time points except at 30 min in the frontal lobe. FA, as one of the most important measures calculated from DTI, has been proven susceptible to microstructural changes in white matter integrity [24]. The FA value was thought to decrease as a result of disruption, disorganization, and loss of myelin sheaths and axonal membranes in white matter [11, 25–27]. In our study, the temporary progressive decrease in FA throughout the frontal lobe 30 min after acute alcohol administration may reflect loss of myelin sheaths induced by ethanol, which will be explained later. The lack of change in FA at other times in the frontal lobe and thalamus may reflect the limitation of diffusion tensor imaging.

MK, defined as the average of the kurtosis over all possible diffusion directions, which is the most important measure calculated from the diffusion kurtosis [13]. The change of MK value depends on the structural complexity of the organization. MK decreased in the frontal lobe and thalamus at 30 min, which suggests a reduction in overall diffusional heterogeneity and is likely due to the following three factors: (1) ethanol can cause local cerebral ischemic and hypoxic injury in the early stage, decreasing the activity of Na^+^, K^+^-ATPase [9]; at the same time, ethanol disturbs intracellular energy metabolism, increasing the influx of Ca^2+^ [28, 29], which jointly leads to cytotoxic edema; (2) it produces massive oxygen radical, has lipid peroxidation, and damages cell membrane [28]; (3) immunoreaction of the organization exists. All the factors mentioned above collaboratively lead to the demyelination, reduce the limitation of medullary sheath on water molecule, and thus decrease MK value.

Unlike FA and MD, the increased MK values, which were observed at the 2 h and 6 h time points in alcohol-administered rats, demonstrate the sensitivity of MK to subsequent complex microstructural change in response to injury. As the illness develops, myelinoclasis, neuronal necrosis, oxidative stress, secondary gliocyte proliferation, and inflammatory cell infiltration may occur in the diseased areas [30–32], which increased the structural complexity of the organization and induced the increase of MK value.

There are several limitations in this study. First, we only chose the frontal lobe and thalamus as ROIs and did not measure AK, RK, AD, and RD. It would be of great interest to examine other regional brain changes including separate brain functional region through DKI metrics. Meantime, we should analyse all DTI and DKI values to figure out more detail of brain microstructure changes after acute alcohol administration. In addition, the sample size used in this study was too small, and we only followed the animals in the acute stage. It would be advantageous to increase the sample size and trace animals through the subacute and chronic phases. Finally, the duration time for the DKI scan was too long. Therefore, future studies will attempt to optimize the scanning parameters.

## 5. Conclusions

Compared with DTI, DKI can provide a more comprehensive evaluation of EtOH-related brain changes at varying time points. Vulnerability of the frontal lobe and thalamus to effects of acute alcohol intoxication suggests that changes in DKI of the frontal lobe and thalamus in vivo may be useful in predicting clinical outcome and facilitating early interventions that might reduce more serious sequelae following acute alcoholism.

## Figures and Tables

**Table 1:** Comparisons of regional DKI and DTI indices, in the frontal lobe and thalamus, between controls and EtOH-administered rats (mean ± SD).

DKI values	Controls	Alcoholism 30 min	Alcoholism 2 h	Alcoholism 6 h
	(*N* = 5)	(*N* = 10)	(*N* = 10)	(*N* = 10)
Frontal lobe				
MK	0.672 ± 0.181	0.375 ± 0.137^*∗∗*^	0.765 ± 0.083^ΔΔ^	0.869 ± 0.065^*∗∗*ΔΔ^
FA	0.257 ± 0.129	0.192 ± 0.042^*∗*^	0.234 ± 0.077	0.194 ± 0.027
MD	0.800 ± 0.086	0.650 ± 0.046^*∗∗*^	0.680 ± 0.092^*∗∗*^	0.617 ± 0.085^*∗∗*^
Thalamus				
MK	0.690 ± 0.181	0.425 ± 0.191^*∗∗*^	1.010 ± 0.187^*∗∗*ΔΔ^	1.161 ± 0.125^*∗∗*ΔΔ^
FA	0.397 ± 0.123	0.345 ± 0.072	0.417 ± 0.070^Δ^	0.390 ± 0.069
MD	0.780 ± 0.048	0.623 ± 0.088^*∗∗*^	0.689 ± 0.095^Δ^	0.628 ± 0.071^*∗∗*^

^*∗*Δ^ *p* < 0.05 was considered to indicate a statistically significant difference.

^*∗∗*ΔΔ^ *p* < 0.01 was considered to indicate a clear statistically significant difference.

^*∗*^Statistical significance was based on comparison with controls.

^Δ^Statistical significance was based on comparison with alcoholism 30 min.
